# Community effectiveness of chloroquine and traditional remedies in the treatment of young children with falciparum malaria in rural Burkina Faso

**DOI:** 10.1186/1475-2875-3-36

**Published:** 2004-10-20

**Authors:** Olaf Mueller, Oliver Razum, Corneille Traore, Bocar Kouyate

**Affiliations:** 1 Department of Tropical Hygiene and Public Health of the Ruprecht-Karls-University Heidelberg, Germany; 2 Centre de Recherche en Santé de Nouna, Burkina Faso

## Abstract

**Background:**

There is little information on the effectiveness of modern compared to traditional malaria treatment from the rural areas of Africa.

**Methods:**

Follow-up of 402 episodes of clinical malaria among pre-school children in Nouna Health District, northwestern Burkina Faso. The exposure of interest was the type of treatment (chloroquine versus traditional); the outcome was clinical response to treatment.

**Results:**

Out of the 402 observed malaria episodes, 87% were treated with chloroquine and 13% with traditional remedies. Overall, community effectiveness was 67% with chloroquine and 54% with traditional treatment. Chloroquine effectiveness was associated with age and ethnicity. An additional interview survey demonstrated wide variations in the dosages of chloroquine given to young children in this community.

**Conclusions:**

The effectiveness of chloroquine, when used within the community, was significantly lower in this study than its known efficacy in the study area. This concerns, in particular, the very young children. These findings demonstrate the need for better education of parents about correct dosage of first-line malaria drugs, and for particular attention in the treatment of very young children.

## Introduction

It has been estimated that at least one million annual malaria deaths occur among young children in rural sub-Saharan Africa (SSA). Most of these deaths are in areas with little access to health services [1-4]. Under such circumstances, home treatment with chloroquine, antipyretics and traditional remedies is the most frequent response of caretakers to fever episodes in children [5]. There is also increasing evidence that improved home management of malaria in young children of SSA can be effective [6,7].

The situation is now complicated by the increasing resistance of Plasmodium falciparum to chloroquine in most SSA countries [8]. Moreover, compared to the efficacy under trial conditions, the community effectiveness (the drug efficacy under real life conditions) of malaria treatment is significantly lower [9]. Chloroquine has been used in Burkina Faso for decades and reported clinical failure rates in children with uncomplicated malaria were around 5% in the early 1990s [10]. However, data from a more recent study on the efficacy of chloroquine in a representative sample of villages in the Nouna Health District in north-western Burkina Faso demonstrated a clinical failure rate of 10% in young children [11]. Nevertheless, chloroquine has remained the official first-line treatment for uncomplicated malaria in Burkina Faso until today.

Traditional treatment for malaria is very common, particularly in the rural areas of SSA, but only few data exist regarding the efficacy of such treatments [5,12]. In rural north-western Burkina Faso, treatment of uncomplicated malaria usually comprises a combination of modern and traditional methods. Chloroquine and antipyretics, often combined with traditional treatments, are the most common community-based treatment regimens for young children during fever episodes [4]. Traditional treatments usually comprise oral and/or skin applications of extracts from eucalyptus plants, acacia, citronella, papaya, guava and the neem tree [13].

This paper reports on the community effectiveness of chloroquine compared to traditional remedies in the treatment of uncomplicated falciparum malaria in young children of rural Burkina Faso.

## Methods

### Study area

The study was conducted in the rural part of the research zone of the Centre de Recherche en Santé de Nouna (CRSN), in Nouna Health District, north-western Burkina Faso. The CRSN study zone comprises 41 villages and Nouna town, with a total population of 55,000. The Nouna area is a dry orchard savanna, populated by subsistance farmers of different ethnic groups. There is a short rainy season which usually lasts from June until October. Malaria is holoendemic but highly seasonal in the study area [14]. Formal health services in the CRSN study zone consist of four village-based health centres and the district hospital in Nouna town [4].

### Study design

This is an observational study at the community level. Data for this study were extracted from the database of a trial on the effects of zinc supplementation on malaria morbidity conducted in the Nouna area in 1999 [14]. During this trial, 709 children aged 6–31 months at enrollment in June 1999 were recruited from 18 villages of Nouna Health District. Study children were followed up until December 1999.

Field methods for data collection have already been described [14]. In brief, the children in the study were regularly visited by village-based field staff for temperature measurement and malaria slide preparation from finger-prick blood in case of fever over the main malaria transmission period (June to December). If fever persisted or recurred, another blood slide was prepared at least one week after the start of the febrile episode. If children were found to have fever or other obvious illness, their caretakers were advised by the field staff to use chloroquine for febrile episodes or to seek diagnosis and treatment at the next governmental health centre. Children found ill during four cross-sectional surveys were treated appropriately by the study physician or referred to Nouna hospital, where they were treated free of charge.

All relevant signs and symptoms manifested by the children were recorded on a standard questionnaire during regular visits of field staff. Moreover, a comprehensive questionnaire was filled in for each disease episode which included information on specific signs and symptoms as well as place, time and type of treatment received. No specific information was recorded on the dosage of chloroquine and on the type of traditional remedies given to the study group.

For this study, only children with a diagnosis of falciparum malaria, defined as an axillary temperature of ≥37.5°C together with a density of ≥5,000 P. falciparum parasites per μl of blood were considered. Inclusion criteria were falciparum malaria treated with chloroquine (with or without antipyretics) but without traditional remedies (group CQ), or with traditional remedies only (group TRAD). Exclusion criteria were a history of febrile illness during the previous four weeks, malaria treatment during the previous four weeks, any treatment with another antimalarial drug during follow up, and missing temperature measurements during the two-weeks follow up period. Multiple episodes of malaria in individual children were considered to be independent with regard to treatment choice [4].

To evaluate treatment outcome, a modified definition of the WHO protocol for assessment of therapeutic efficacy of antimalarial drugs in areas with intense transmission was used [15]. Treatment failure (TF) was defined as development of severe malaria on day 1–14 or fever recurrence (axillary temperature ≥37.5°C) on day 3–14. Adequate clinical response (AR) was defined as the absence of fever on day 3–14, without meeting any of the criteria of TF.

### Chloroquine dosage survey

To retrospectively estimate the chloroquine dosages usually given to febrile young children in the study area, 55 individual interviews with randomly selected mothers of young children from three villages in the study area were conducted during a malaria survey at the end of the 2001 rainy season. The villages were purposely selected to represent the rural study population in its socio-cultural, demographic and geographical diversity. Questions concerned the kind of treatment (western and/or traditional) and the dosages of western drugs used during treatment of the last febrile episode of survey children, the current availability of chloroquine in the household and the origin of chloroquine drugs. The weight of children was measured with a Salter hanging spring scale.

### Laboratory procedures

Blood films were kept in closed slide boxes until they were transported to Nouna (two to three times per week). They were Giemsa-stained at the Nouna hospital laboratory and transported afterwards to the Centre National de Recherche et de Formation sur le Paludisme (CNRFP) in Ouagadougou for reading. All films were examined by two experienced laboratory technicians using a ×100 oil immersion lens and ×10 eyepieces. In case of significant discrepancy between the results of the two technicians, blood slides were read by a third investigator. Blood films were analysed for the species-specific parasite density per μl by counting against 500 white blood cells and multiplying by sixteen (assuming 8,000 white blood cells per μl of blood). Slides were declared negative if no parasites were seen in 400 fields on the thick film.

### Statistical analysis

Data were entered at the data management department of the CRSN into a data bank (Microsoft Access, version 97). All questionnaires were checked by supervisors before computer entry. Parasitological data were entered into EpiInfo (version 6.0) at the CNRFP, and the data were transferred to the CRSN. All data were checked for range and consistency before data entry.

The outcome of malaria episodes (AR vs. TF) was assessed dependent on the treatment given by the caretakers. Episodes in which the child had received at least one dose of chloroquine were grouped in the treatment group CQ, and episodes in which caretakers had given only traditional treatment in the treatment group TRAD. The hypothesis was tested that the samples in the CQ and the TRAD treatment groups were from populations with the same distribution of baseline variables using the Wilcoxon ranksum test for continuous variables and the chi-squared test for categorical variables. Community effectiveness of treatment with chloroquine was assessed both by intention to treat (i.e. adherence to advice by the field workers to give chloroquine) and considering only those cases who actually complied. A stratified analysis by age bands was started, assuming that age would be an important determinant of treatment outcome. Logistic regression modelling was then used to control for differences in the distribution of baseline characteristics such as age, sex and parasite density in the two treatment groups. Stata 7.0 statistical software was used for all calculations.

### Ethical aspects

Approval for the RCT from which the data used were drawn was granted by the Ethical Committee of the Heidelberg University Medical School and the Ministry of Health in Burkina Faso.

## Results

### Follow-up study

A total of 330 children (175 boys and 155 girls), who contributed 402 episodes of falciparum malaria, were followed up. Most of the episodes (65.7%) occurred between August and October (Table 1).

**Table 1:** Distribution of febrile episodes by treatment type over the main malaria transmission period in young children of rural Burkina Faso

	Month					
	
	7/99	8/99	9/99	10/99	11/99	total
Group CQ	25	126	108	75	16	350
Group TRAD	1	15	15	19	2	52
Total	26	141	123	94	18	402

In 350 (87.1%) of the episodes, the child had received at least one dose of chloroquine (treatment group CQ); in 52 episodes (12.9%), the caretakers had given only traditional treatment (treatment group TRAD). Table 2 shows the distribution of baseline characteristics in the two treatment groups. The median parasite density was significantly lower in the TRAD group (p = 0.001), but diarrhoea was observed in a larger proportion of episodes in this group than in the CQ group (p = 0.04). In the Bwaba ethnic group, 33.3% of malaria episodes had been treated with traditional treatment, compared to 8.7% in the other ethnic groups (p < 0.0001).

**Table 2:** Baseline characteristics of malaria episodes in study children in rural Burkina Faso

	Group CQ (n = 350)	Group TRAD (n = 52)	p-value *
Male/female	183/167	30/22	0.47
Median age, range (months)	20 (8 – 35)	19 (9 – 33)	0.36
Median parasite density/μl, D0 Range	26 220 5000 – 1 140 000	16 352 5000 – 86 656	0.001
Median temperature D0 (°C)	38.2	38.2	0.72
Diarrhoea D0 (%)	64/350 (18.3)	16/52 (30.8)	0.04
Vomiting D0 (%)	39/350 (11.1)	3/52 (5.8)	0.24
Ethnicity			<0.0001
Marka	180	16	
Mossi	72	8	
Bwaba	46	23	
Peulh	44	5	
Others	8	0	

* Chi-squared test for categorical variables, ranksum test for continuous variables

Group CQ: received at least one dose of chloroquine

Group TRAD: received only traditional treatment

D0: Day of onset of malaria episode

The caretakers had been advised to give chloroquine to all children participating in this study. Compliance with this advice, stratified by age group, is shown in Table 3. Compliance was independent of sex (data not shown), except in the age group 15–21 months, in which 98.2% of episodes in girls, but only 86.8% of episodes in boys, were treated with chloroquine (p = 0.02). Community effectiveness as a result of following the advice to give chloroquine, measured by the proportion of episodes in which the child had an AR was 64.9% (95% CI: 60.0–69.6%). This proportion did not increase appreciably when we restricted the analysis to episodes in which the child had actually received chloroquine (Table 3). In the TRAD group, children had an AR in 53.9% of the episodes. Our study did not have sufficient power to show a difference between the TRAD and the CQ groups as small as the one observed (-11%) at the 5% significance level. In the CQ group, outcome was strongly and positively associated with age, as demonstrated by the chi-squared test for linear trend (p = 0.014). A comparable result was not shown in the TRAD group, possibly because of small case numbers.

**Table 3:** Compliance with treatment and outcome of treatment, stratified by age group

	Compliance	Outcome of treatment		
Age group	CQ given n (%)	AR, total n (%)	AR, CQ group n (%)	AR, TRAD group n (%)

8 – 14 months	87 (81.3)	58 (54.2)	49 (56.3)	9 (45.0)
15 – 21 months	121 (91.7)	89 (67.4)	83 (68.6)	6 (54.6)
22 – 28 months	86 (87.8)	63 (64.3)	57 (66.3)	6 (50.0)
29 – 35 months	56 (86.2)	51 (78.5)	44 (78.6)	7 (77.7)
Total	350 (87.1)	261 (64.9)	233 (66.6)	28 (53.9)
CI a	-	60.0 – 69.6%	61.4 – 71.5%	39.5 – 67.8%
p-value	0.13 b	0.004 c	0.014 c	0.17 c

a CI: Binomial exact 95% confidence interval

b p-value: Chi-squared test for heterogeneity (within each column)

c p-value trend: Chi-squared test for linear trend (within each column)

CQ group: Received at least one dose of chloroquine

AR: Adeaquate clinical response

The crude odds ratio for TF in the CQ group vs. the TRAD group was 0.59 with a 95% confidence interval that included unity (Model 1 in Table 4). Logistic regression modelling was used to assess the degree to which the observed advantage of chloroquine treatment over traditional treatment was due to a different distribution of baseline characteristics in the two treatment groups. Inclusion of the variables age group (with four seven-month age bands) and sex (Model 2 in Table 4), as well as parasite density (log-transformed and grouped in three bands) and diarrhoea on day 0 (Model 3 in Table 4) did not change the odds ratio much; if there was any advantage of chloroquine over traditional treatment it was slightly attenuated. However, removing all episodes among children of the Bwaba ethnic group from the analysis decreased the odds ratio, and the corresponding confidence interval no longer included unity (Model 4 in Table 4). This means that in the other ethnic groups, chloroquine treatment carried only 0.43 (95% CI: 0.19–0.91) times the odds of treatment failure of traditional treatment and thus conveyed a significant advantage.

**Table 4:** Regression modelling of treatment failure, CQ vs. TRAD group

Model	Variables	Odds Ratio (95% CI)	p-value
1	crude	0.59 (0.33 – 1.06)	0.08
2	age group, sex	0.61 (0.34 – 1.11)	0.10
3	age group, sex, parasite density, diarrhoea	0.64 (0.35 – 1.17)	0.15
4	age group, sex, parasite density, diarrhoea excluding Bwaba ethnicity	0.43 (0.19 – 0.97)	0.04

CQ group: received at least one dose of chloroquine

TRA group: received only traditional treatment

Table 2 already showed evidence of an interaction between treatment pattern and ethnic group. Therefore, a subgroup analysis was done in which additional differences between the Bwaba and the other ethnic groups were found. First, among the other ethnic groups, children who received chloroquine had a significantly higher log-transformed mean parasite density than those who received traditional treatment (p = 0.002). The difference in parasite density was smaller and not significant among the Bwaba (p = 0.26). Secondly, treatment outcome was not significantly associated with age group among the Bwaba, while it was so among the other ethnic groups. In Model 4, an increase in age by one age band (equivalent to seven months) was associated with an almost 30% reduction in the odds of treatment failure (p = 0.004) after control for treatment type, sex, parasite density and diarrhoea on day 0 (also see Table 3). With a number of important factors determining treatment type and/or outcome varying by ethnic group, the stratified analyses were warranted.

### Chloroquine dosage survey

A total of 55 mothers participated in the survey with 28 girls and 27 boys aged 3–27 months (median: 14 months). During the most recent fever episodes of respective children, home treatment with chloroquine was reported in 32/55 (58%) (8/32 in combination with paracetamol and 1/32 in combination with traditional treatment), treatment at the local health centre in 15/55 (27%), traditional treatment in 6/55 (11%), and only paracetamol treatment in 2/55 (4%). The mean weight of chloroquine-treated children was 8.5 kg (range 3.2 – 12.0 kg). The mean (median) total dose of chloroquine used was 34 mg/kg and 25 mg/kg respectively (range 5–110 mg/kg) (figure 1). Only 9/55 (13%) mothers reported current availability of chloroquine tablets in their household (median 3 tablets, range 1–10). Most chloroquine drugs were purchased from local shops and drug sellers.

## Discussion

The main finding of this study is a rather low community effectiveness of chloroquine in the treatment of malaria episodes among young children in rural Burkina Faso. Overall, a failure rate of 35% was observed which declined only marginally to 33% when malaria episodes in which caretakers did not comply with the advice to give chloroquine were excluded. This is considerably higher than the 10% treatment failure rate observed in a study on chloroquine efficacy in young children of the area [11]. These findings support the hypothesis that treatment efficacy declines drastically under real life conditions in the community and demonstrate that efficacy trials need to be complemented with observational studies to assess the actual health benefits conferred by an intervention [9,16].

The effectiveness of treatment with chloroquine increased significantly and almost linearly with age, even after controlling for parasite density. The most likely explanation is a rapid development of immunity within the age range of our study children, as has been observed in other studies [1,17,18]. This implies that particular care is needed in the treatment of the youngest children in whom immunity will be weakest. Given the fact that the children who participated in the study on chloroquine efficacy in the Nouna area were in the most susceptible age group for developing clinical malaria (mean age 10 months, range 6–15), the loss of efficacy under community conditions observed in this study becomes even more pronounced [11].

Only a small number of episodes were treated solely with traditional treatment, possibly a consequence of the view of the local population that western malaria treatment is more effective than traditional treatment [13]. This may not apply to all strata of the population, as there was an interaction between type of treatment and ethnicity. Moreover, while children receiving traditional treatment had a higher risk of treatment failure than children treated with chloroquine after control for age, sex and parasite density, this association did not reach statistical significance until episodes of children belonging to the Bwaba ethnic group were removed from the analysis. Interestingly, crude failure rates were not higher among cases from the Bwaba group compared to those belonging to other ethnic groups. Also these results support the findings from another study in Burkina Faso which has also shown a lower effectiveness of traditional compared to modern malaria treatment, traditional treatments are not identical in different populations [17]. Thus, it may well be that the traditional treatment used in the Bwaba ethnic group is more effective against malaria compared to other traditional treatments in the area. Finally, the observed significant association of traditional treatment with diarrhoea on day 0 may well be a chance finding, given the lack of a difference between treatment groups in all other clinical and parasitological parameters.

This study has two limitations. First, information on the dosage of chloroquine and on the nature of traditional remedies given to children in the study was lacking. However, data from the interview survey provide a good estimate of chloroquine dosages used. Although these data demonstrate a wide variation of chloroquine dosages, it is reassuring that the reported median dosage complies with the national recommendations of a total dose of 25 mg/kg chloroquine for uncomplicated malaria. So why is there such a difference between efficacy and effectiveness? The reason may well be a combination of partly chloroquine underdosing, poor quality of drugs purchased from local shops and drug sellers and high frequency of vomiting in febrile young children [19-23]. Moreover, the possibility of a significant reporting bias during interview surveys also has to be taken into consideration. Secondly, a purely clinical definition of treatment failure was used. Intercurrent febrile illnesses may thus have been misclassified as persisting malaria, thereby overestimating failure rates. However, this is likely to have only a small effect, since the fraction of febrile episodes attributable to malaria (using a highly specific definition) in this cohort of children was found to be above 50 percent [24].

In conclusion, this study provides clear evidence for a rather low and age-dependant community effectiveness of chloroquine in the treatment of P. falciparum malaria in an area of rural Burkina Faso where the efficacy of chloroquine is still sufficiently high in use as first-line treatment. The observed discrepancy between efficacy and community effectiveness implies that many children received insufficient doses of chloroquine. Our findings thus point to the importance of (1) better education of parents on correct dosage of first-line malaria drugs, as well as on danger signs requiring a visit at a health centre, (2) quality-control of all malaria drugs used in the community, and (3) particular care in the treatment of immunologically naïve very young children. Finally, more studies on the types and effects of traditional treatments used in different population groups are needed.
